# ADAMTS Proteases: Their Multifaceted Role in the Regulation of Cancer Metastasis

**DOI:** 10.54457/DR.202401004

**Published:** 2024

**Authors:** Rachele Bacchetti, Shengnan Yuan, Elena Rainero

**Affiliations:** 1 School of Biosciences, Department of Biomedical science, Firth Court, Western Bank, Sheffield S10 2TN, UK

**Keywords:** ADAMTS, Extracellular matrix, Cancer, Tumour microenvironment, Proteases, Metastasis and cell signalling

## Abstract

Cancer leads to nearly 10 million deaths worldwide per year. The tumour microenvironment (TME) is fundamental for tumour growth and progression. A key component of the TME, the extracellular matrix (ECM) has recently become a focus of interest in cancer research. Dysregulation of ECM synthesis and proteolysis leads to uncontrolled tumour growth and metastasis. Matrix remodelling enzymes, secreted by cancer cells and stromal cells, modify the overall structure and organisation of ECM proteins, therefore influencing biochemical interactions, tissue integrity and tissue turnover. While A Disintegrin and Metalloproteinases (ADAMs)’ and matrix metalloproteinases’ role in cancer has been deeply investigated, other proteolytic enzymes, like ADAMs with thrombospondin(-like) motifs (ADAMTSs) have been gaining interest due to their roles in modulating cancer cell-ECM interactions and oncogenic signalling pathways. In this review, we will discuss the dysregulation of ADAMTSs in cancer and their roles in regulating cancer development and progression, via ECM remodelling and cell signalling modulation.

## Introduction

The tumour microenvironment (TME) critically influences tumour growth and metastasis. It is composed of a variety of cell types, including tumour cells, stromal cells, blood vessels and inflammatory cells. In addition, the extracellular matrix (ECM) is an essential non-cellular component of the TME, which supports cell-cell and cell-matrix crosstalk under both normal and pathological conditions. During tumorigenesis, the ECM can modulate all the hallmarks of cancer^[1]^ via both altered organisation and expression of ECM proteins, such as collagens, and altered remodelling, mediated by ECM degrading enzymes. Several protease families have been implicated in ECM degradation. The function of matrix-metalloproteinases (MMPs) and A disintegrin and metalloproteinases (ADAMs) has been extensively characterised in tumorigenesis. Indeed, dysregulation of these proteases was found to promote tumour progression by supporting ECM remodelling, angiogenesis, cancer cell migration and invasion, and disruption of cancer-associated immune reactions^[2,3]^. Since these proteases have been found relevant to the progression of several types of cancer, they were considered as potential targets for cancer diagnosis and therapy. Additionally, the tissue inhibitors of metalloproteinases (TIMPs) were exploited for therapeutic purposes in cancer, due to their ability to inhibit the catalytic activities of MMPs and ADAMs^[4]^. This review will consider the family of metalloproteinases a disintegrin and metalloproteinase with thrombospondin(-like) motifs (ADAMTS) and its roles in cancer biology.

**Fig. 1:**
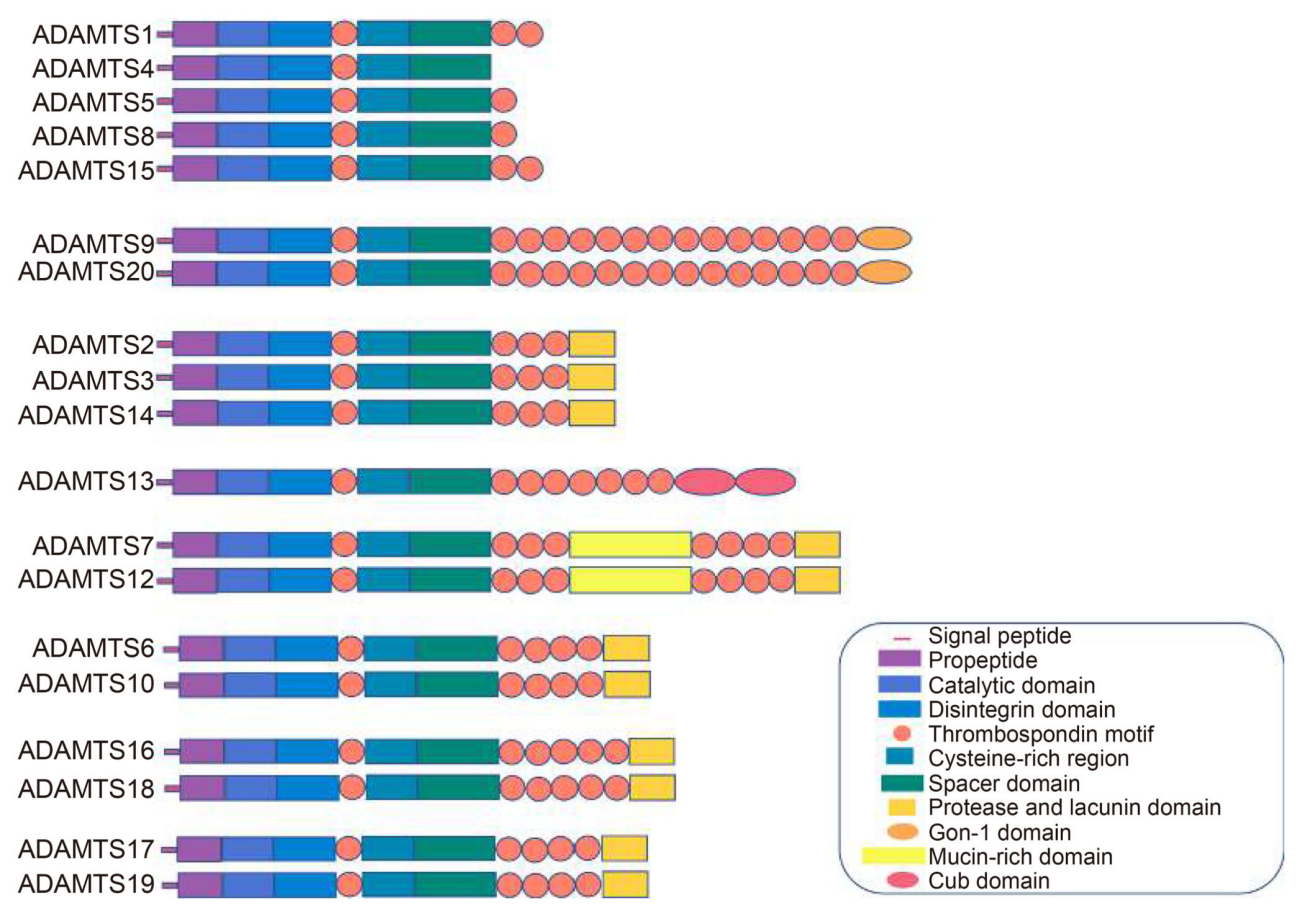
ADAMTS Family. Schematic representation of the structure of the 19 different ADAMTS family members. They all share the same domain organization and some members have have unique domains at their C-terminus. The diagram is not to scale. Figure adapted from ^[7]^.

ADAMTS family consists of 19 secreted enzymes that can have one of or more thrombospondin motifs. Since Kuno’s first identification of an ADAMTS member^[5]^, they have been studied for their catalytic activity in different diseases, while over the last decade the research interest in their involvement in cancer has increased. Their structure and function have been extensively reviewed^[6,7]^. All ADAMTS members share the same domain organisation. Starting from the N-terminus, they contain a signal-peptide, a pro-region, a metalloproteinase domain, a disintegrin-like domain, a central thrombospondin type 1 motif, a cysteine rich domain, and a spacer region. All ADAMTSs, except ADAMTS4, have multiple thrombospondin type 1-like motif (TSR-1) following the spacer region and some will have unique domains at their C-terminus. These ancillary domains are thought to modulate ADAMTS activity, substrate specificity and tissue localisation, which is why structurally similar ADAMTSs could show similar catalytic activity (Fig. 1). ADAMTS1, 4, 5, 8, 9, 15 and 20 have been classified as proteoglycanases, ADAMTS2, 3 and 14 as propeptidases, ADAMTS13 as von Willebrand factor-cleaving protease, ADAMTS7 and 12 as Cartilage oligomeric matrix protein proteinases, ADAMTS16 and 18 has been recently reported to cleave fibronectin (FN)^[8,9]^ while the substrate of ADAMTS6, 17 and 19 have not been defined yet. Activation of ADAMTS catalytic activity varies across the family. Most ADAMTSs, like ADAMTS4 and 5, become activated by cleavage of the pro-domain. Others, like ADAMTS13 and ADAMTS7, can cleave specific substrates with their pro-domain intact^[6]^. It is these variety in substrate specification, tissue localisation, activation and inhibition^[7]^ that, interestingly, allows ADAMTSs to have both tumour promoting and tumour suppressing roles depending on the cancer type.

ADAMTSs play an important role in embryonic development, as different family members have been shown to control palate closure, interdigital web regression, cardiac development and morphogenesis, eye development, ciliogenesis, skin pigmentation and connective tissue turnover^[10–16]^. In addition, several recessive genetic disorders are associated with mutations in ADAMTS genes, as reviewed in^[10]^. The role of ADAMTSs has been extensively studied in osteoarthritis, a disease characterised by bone inflammation, leading to degeneration and loss of cartilage function. In this context, different members of the ADAMTS family have been shown to contribute to ECM remodelling, cartilage degeneration and synovial inflammation^[17]^. In this review, we will summarise the changes in ADAMTS expression associated with cancer progression and will explore the molecular mechanisms through which ADAMTSs can control tumour progression, by focusing on their role in directly modulating the TME and the ECM and indirectly controlling key signalling pathways, predominantly by the modulation of growth factor availability.

### ADAMTS expression is deregulated in cancer

**Fig. 2:**
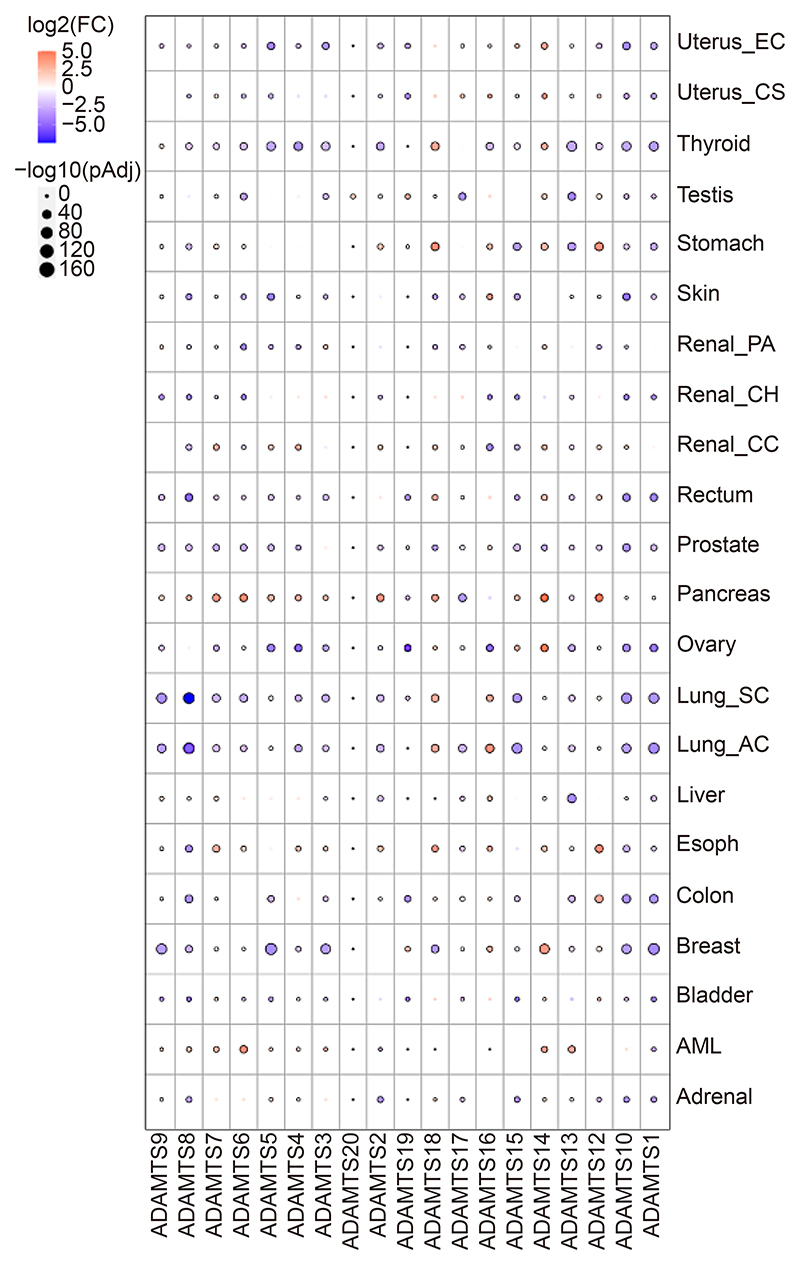
ADAMTS Pan-cancer analysis. RNAseq expression values of ADAMTS family members in different tumour types and the corresponding healthy tissues were collected from online databases (GEO, GTex, TCGA and TARGET) and were analysed using TNM plotter^[10]^. Log2 fold changes are presented, the size of the dot represents the p value.

To provide a general overview of the changes in ADAMTS expression in cancer, we performed a pan-cancer analysis using TNM plotter (Fig. 2) ^[18]^. Red represents upregulation and blue represents downregulation in tumours compared to the corresponding healthy tissue. The biggest changes in ADAMTS expression are observed in breast (BC), lung (LC), ovarian (OC), pancreatic (PDAC) and thyroid cancer. Strikingly, while most ADAMTS family members are upregulated in PDAC, there seems to be a differential regulation of different ADAMTSs in most cancertypes. Moreover, ADAMTS14 and 18 are upregulated in most cancers, while ADAMTS1, 8, 9, 10 and 15 are downregulated across the board. ADAMTS20 does not appear to be differentially regulated in any cancer.

Interestingly, most family members are downregulated in tumours, compared to normal tissues (Fig. 2). Evidence suggests that this downregulation is predominantly due to promoter hypermethylation. Indeed, the expression of ADAMTS8, 9, 12, 16, 18 and 19 was found to be reduced through methylation in the CpG islands in the promoter region in LC, BC, gastric (GC), colorectal (CRC), PDAC, prostate cancer (PC) and renal cell carcinoma (RCC)^[19–28]^. Treatment with a DNA methyltransferase (DNMT) inhibitor or the demethylating agent 5-aza-2 ′ -deoxycytidine (ADC) restored ADAMTS8, 9, 12 and 18 expression^[19,20,22,23,29,30]^. ADAMTS8 levels were also restored upon double knockout of DNMT1 and DNMT3B in CRC cells^[22,23]^. Moreover, in GC cells, DNMT3A was identified as the primary methyltransferase mediating ADAMTS9 promoter hypermethylation, as decreased expression of DNMT3A increased ADAMTS9 expression. Mechanistically, the ubiquitin ligase ring finger protein 180 (RNF180) promoted DNMT3A proteasomal degradation. RNF180 is a tumour suppressor found downregulated in GC. Therefore, in the absence of RNF180, DNMT3A is upregulated and suppresses ADAMTS9 expression^[20]^. These data suggest that different methyltransferases are likely to regulate the expression of different ADAMTS members and/or they are cancer type specific. Further exploration of the mechanisms behind the hypermethylation and downregulation of ADAMTSs is therefore needed. Interestingly, there was a significant association between ADAMTS8 methylation status and GC lymph node metastasis^[19]^. As two DN-MT inhibitors have been approved by the Food and Drug Administration^[31]^, it would be interesting to test whether these could be effective in cancer models, such as LC, BC, GC, CRC and PDAC, whether the inactivation of several members of the ADAMTS family has been shown to occur through promoter methylation.

In addition, ADAMTS8 expression was reported to be regulated by the transcription factor GATA1. GATA1 expression was found significantly downregulated in LC samples compared to healthy tissues and this correlated with reduction in ADAMTS8 levels^[32]^.

Other than promoter methylation, a group of small non-coding RNA termed microRNAs (miRNAs) was also found to regulate ADAMTS expression post-transcriptionally in BC. This occurs through binding to the mRNA and translation inhibition^[33]^. miR-365 was found to directly suppress ADAMTS1 expression. BC cell lines treated with miR-365 mimics showed significantly reduced ADAMTS1 levels, while cells treated with miR-365 inhibitors showed significantly increased ADAMTS1 expression. Introducing miR-365 in BC cells overexpressing ADAMTS1 restored the proliferation and invasion ability which was inhibited by ADAMTS1^[34]^. In contrast, miR-365 was reported to be downregulated in BC tissues and triple negative BC cell lines, compared to pre-cancerous samples and non-transformed mammary epithelial cells^[35]^. In this context, miR-365 overexpression suppressed cell proliferation, migration and invasion, through the inhibition of ADAM10 expression^[35]^. More research is needed to clarify the reason behind these inconsistent results. Similarly, miR-221-3p, found upregulated in several cancer types, was found to suppress the expression of ADAMTS6 in MCF-7 cells^[36]^.

Despite the fact that ADAMTS8 has been mainly reported to be downregulated in cancer, BC patients with low levels of ADAMTS15, in combination with high ADAMTS8 expression, showed the worst prolonged relapse-free survival^[37]^. Both proteases are components of the proteoglycanase subfamily, sharing high sequence similarity, suggesting that catalytic-independent roles of the proteases might play a role in this context. Similarly, ADAMTS12 and ADAMTS2 could potentially be co-regulated in GC, where both have been reported to be upregulated^[38]^. These proteases belong to different ADAMTS subfamilies, suggesting that they might play complementary roles in driving tumour progression. This suggests a close interplay between different ADAMTS family members, which warrant further research.

Immunohistochemical analysis of normal and cancerous tissues in CRC patients showed a decrease in ADAMTS15 expression in tumours compared to normal. In addition, ADAMTS15 was found to be mutated in a small set of CRCs. In particular, the G489fs mutation results in a protein that lacks the last two thrombospondin-1 motifs. This mutation was shown to compromise the plasma membrane association of ADAMTS15, as only the G489fs form was found secreted in the conditioned media and associated with the ECM. Interestingly, in vitro studies showed reduced colony formation and cell invasion when wild-type ADAMTS15 was expressed in CRC cells compared to G489fs, suggesting that the mutation abrogates the tumour suppressive functions of ADAMTS15^[39]^. Further work is required to determine the molecular basis behind ADAMTS15 tumour suppressive role in CRC, and whether this is dependent on its aggrecanase activity.

Other ADAMTS members have been found deregulated in cancer patients, but no regulation mechanism has been identified. ADAMTS1 and 5 expression level was found significantly increased in malignant and borderline ovarian tumour samples compared to benign tumours, suggesting they might play a role in OC metastasis^[40]^. Likewise, high ADAMTS5 expression levels in CRC patients are correlated to cancer lymphatic invasion and lymphatic metastasis^[41]^, suggesting a link between ADAMTS5 and CRC progression. In PC, mRNA levels of ADAMTS1, 8 and 9 were found to be reduced in prostatic carcinoma cell lines (PC3, DU145 and LNCaP) compared to prostatic stromal cells from benign prostatic hyperplasia patients (BPH), while ADAMTS15 mRNA expression was higher in carcinoma cells and ADAMTS4 and 5 were not detectable in cancer cells but present in BPH samples^[42]^.

**Fig. 3:**
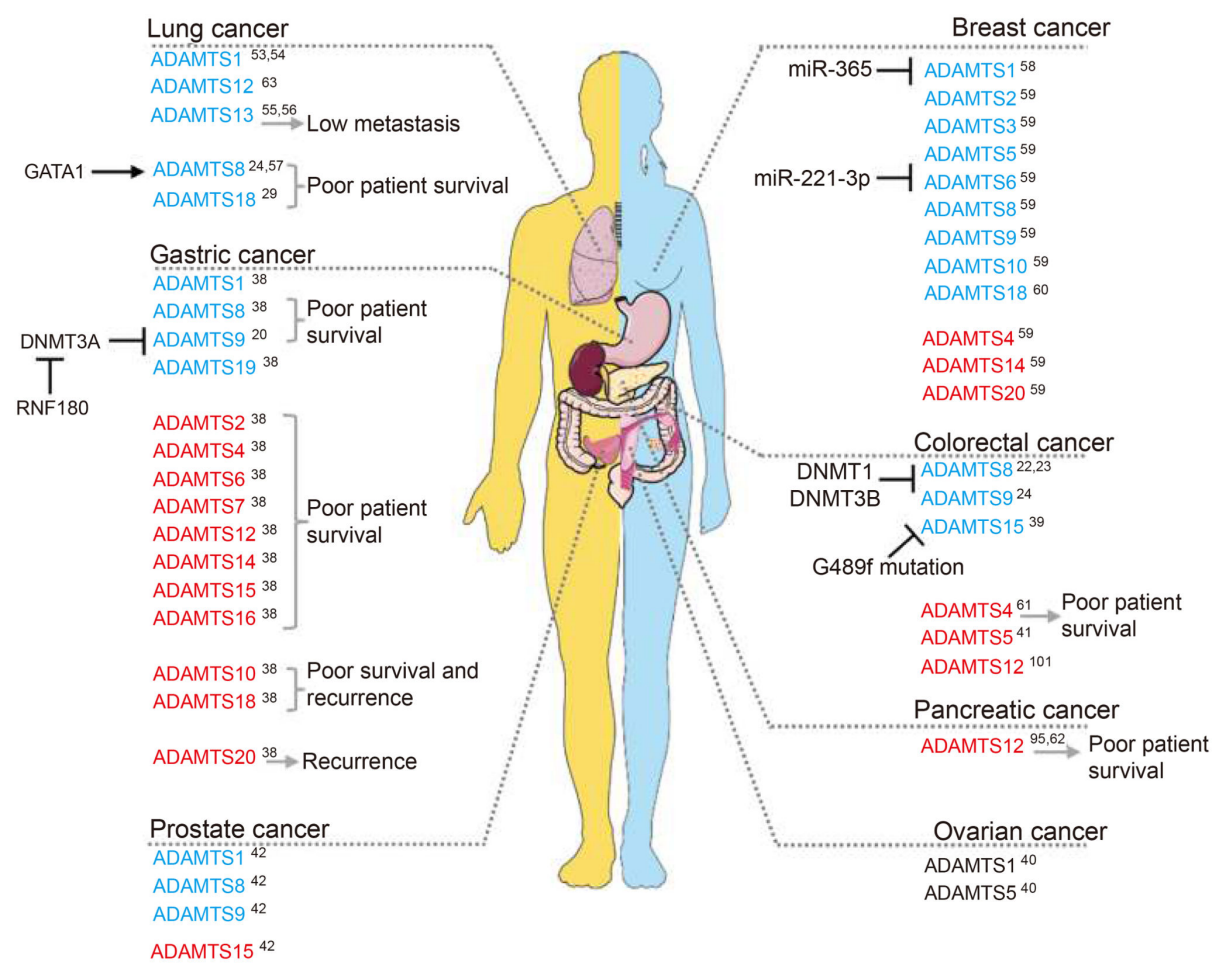
ADAMTSs expression in cancer. Schematic representation of the deregulation of ADAMTS family member expression in different cancer types. Upregulated ADAMTSs are marked in red and downregulated ADAMTS are marked in blue^[47–56, 57]^. DNMT, DNA methyltransferase. Image generated with elements from Servier Medical Art, licensed under CC BY 4.0.

Data from the literature mostly corroborate our RNA-seq analysis. Indeed, ADAMTS are mostly downregulated in PC, BC, CRC and LC, while they are upregulated in PDAC (Fig. 3). Overall, ADAMTS proteases are dysregulated in a variety of ways depending on the cancer type. Importantly, ADAMTSs can be detected in body fluids and could therefore represent novel biomarkers. Indeed, ADAMTS7 was detected in the urine of bladder cancer patients^[43]^; while reduced serum levels of ADAMTS18 correlated with high tumour stage, positive lymph node metastasis, distant metastasis and shorter overall survival in cervical cancer patients^[44]^. Upregulation of cell-free ADAMTS8 mRNA was detected in the plasma from LC patients, compared to healthy controls^[45]^. Finally, targeted proteomics was used to identify an ADAMTS15-containing serum protein signature in resectable GC patients^[46]^. As most of the data are correlations between ADAMTS expression and patient outcomes, more mechanistic studies are needed to demonstrate a causative role of ADAMTS expression/downregulation in driving cancer progression.

### ADAMTSs modulate the TME to control tumorigenesis

The ECM provides both biochemical and structural support for cells within tissues, being responsible for cell-cell communication, adhesion and proliferation^[58]^. While during early tumour progression the ECM can function as a physical barrier that encapsulates the primary tumour mass, preventing tumour growth, at later stages the ECM plays a tumour promoting role, supporting all the hallmarks of cancer^[1]^. The ECM is composed of a variety of proteins, including proteoglycans (versican, brevian, neurocan and aggrecan), collagens and glycoproteins (such as FN, vitronectin and laminin). The core matrisome is formed by over 300 proteins, as defined in the matrisome project^[59,60]^. Cancer cells and cancer associated fibroblasts (CAFs) can secrete matrix-degrading enzymes. ECM degradation and remodelling by these enzymes favour cell migration and invasion, leading to cancer progression. Proteases, MMPs, plasmin and cathepsins, among others, are ECM-degrading enzymes that are able to cleave different ECM substrates like collagens, FN, and laminins^[61]^. Moreover, cleaved ECM fragments, known as ‘matrikines’ can function as signalling molecules to support multiple cell behaviours, including proliferation, migration and protein synthesis^[62]^. Proteases are normally fine-tuned by their endogenous inhibitors, such as TIMPs and α2-macroglobulin to maintain homeostasis, but deregulation of this process often occurs during pathogenesis^[63]^. In this section, we highlight how deregulated activity of ADAMTS family members (both in cancer and stromal cells) impacts on tumour development by modulating ECM component degradation and cell-ECM interaction.

### ADAMTS-dependent ECM remodelling in cancer progression

**Fig. 4:**
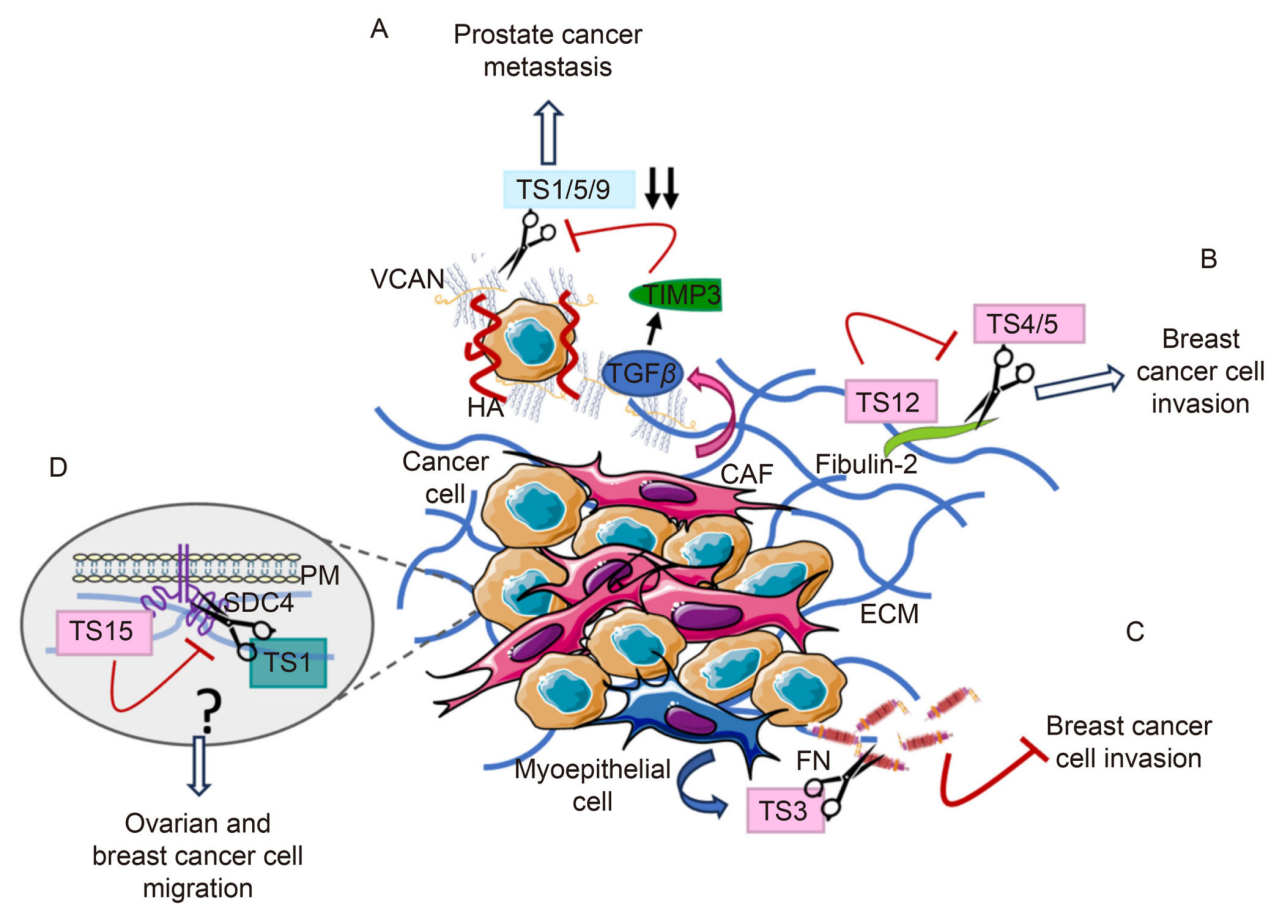
ADAMTSs modulation of the TME. A. In PC, CAF secreted TGFβ enhances TIMP3 leading to inhibition of ADAMTS1, 5 and 9 and accumulation of versican (VCAN). Versican and hyaluronic acid (HA) for a pericellular matrix that encapsulates PC cells further enhancing cancer metastasis. B. In BC, ADAMTS4 and 5 cleavage of fibulin-2 leads to higher cell invasive phenotype, in both BC cells and fibroblasts. This is inhibited by ADAMTS12 binding to fibulin-2. C. ADAMTS3 secreted by myoepithelial cells inhibits BC cell invasion through cleavage of fibronectin (FN). D. ADAMTS1 was found to cleave syndecan-4 (SDCN4) in OC promoting invasion. ADAMTS15 increases cell membrane expression of syndecan-4, in BC, potentially by inhibiting ADAMTS1. ADAMTSs have been colour coded based on cancer type, light blue for PC; pink for BC; and teal for OC. Image generated with elements from Servier Medical Art, licensed under CC BY 4.0.

PC stroma is enriched in hyaluronan and versican, and this is associated with metastatic progression. *In vitro*, highly metastatic PC cell lines PC3 and DU145 treated with prostate fibroblast conditioned media containing versican, aggrecan and hyaluronan were observed to assemble and rearrange a pericellular matrix, which promoted cell motility^[64]^. Interestingly, versican deposition has been shown to promote cell migration by preventing cell adhesion to FN, therefore driving cell motility^[65]^. More recently, ADAMTS9-dependent versican cleavage has been found to modulate smooth muscle cell adhesion to the ECM^[66]^. These data suggest a potential mechanism through which changes in ECM composition might drive PC cells invasion, leading to metastatic spread. In this context, we can envisage that loss of expression of ADAMTSs responsible for versican cleavage might foster versican accumulation and therefore cell migration. Consistent with this, ADAMTS1 was reported to be downregulated in PC (Fig. 2 and 3). Moreover, stromal expression of ADAMTS1, 5, 9 was almost completely lost, while the expression of TIMP-3 and versican was upregulated, in response to elevated transforming growth factor beta (TGFβ) stimulation *in vitro*^[42]^. This suggests that elevated versican deposition observed in PC could be due to the upregulation of TIMP-3 and the concomitant downregulation of ADAMTSs, leading to reduced ADAMTS proteolytic activity, therefore leading to versican accumulation (Fig. 4A). It would therefore be interesting to directly test the role of ADAMTSs in promoting versican accumulation and assess how ADAMTS inhibition impinges on versican-driven PC cell migration *in vitro* and *in vivo*. Furthermore, TGFβ1 has been shown to promote versican secretion from stromal cells^[42]^, while stromal accumulation of versican in tumours has been associated with increased inflammation^[67]^. Therefore, ADAMTS-dependent regulation of versican levels could potentially impact on the immune regulation of the TME.

Similarly, versican was found upregulated in OC stroma and high expression correlated with poorer overall and progression-free survival, as well as chemotherapy resistance. *In vitro*, stimulation of OC cells with versican-containing conditioned media seemed to increase cell invasion^[68]^. The role of versican cleavage has not been investigated in this work, but it would be interesting to determine whether (a) intact versican is mediating the pro-tumorigenic effect, and therefore ADAMTs would be tumour suppressor in this context, or (b) versican cleavage is required, meaning that ADAMTSs might be modulating versican activity. Consistent with the latter hypothesis, versican cleavage product versikine has been suggested to play a role in cancer inflammation^[69]^. Indeed, while intact versican is mostly immunosuppressive, versikine has been shown to promote T cell infiltration and conventional dendritic cell generation in the peritumoral stroma in LC, overcoming resistance to immunotherapy^[70]^.

ADAMTS5, and to a lesser extent ADAMTS4, was identified to modulate BC cell migration through the cleavage of the glycoprotein fibulin-2^[71]^. In particular, while the overexpression of fibulin-2 prevented BC cell invasion, the co-expression of fibulin-2 and ADAMTS4 and 5 strongly promoted the invasive phenotype. Interestingly, conditioned media from BC cells overexpressing both fibulin-2 and ADAMTS5 strongly promoted fibroblast activation and invasion, suggesting a broader role of ADAMTS5 in shaping the TME to potentially drive metastasis^[71]^. Remarkably, ADAMTS12 was found to bind to fibulin-2 and protect it from being degraded by ADAMTS4 and 5, showing a tumour suppressive behaviour^[72]^. Consistently, the expression of ADAMTS12 prevented ADAMTS5-induced invasion *in vitro*^[71]^ and opposed tumour growth *in vivo* in xenograft experiments in nude mice^[72]^. These observations raise the intriguing hypothesis that the interplay between different ADAMTS family members contributes to both controlling the invasive abilities of cancer cells and shaping the stroma in BC, leading to the formation of a pro-tumorigenic TME (Fig. 4B). It will also be interesting to investigate the interplay between different ADAMTS substrates, as versican is also overexpressed in BC^[73]^ and its cleavage by ADAMTSs could play a role also in BC progression.

In OC patients, mutations in ADAMTS family members (1, 6, 9, 13, 15, 16 and 18) have been associated with platinum-based chemotherapy sensitivity^[74]^. Among them, ADAMTS16 mutation is the most frequent. ADAMTS16 missense mutations located on the TSR-1, ADAM Spacer 1, protease and lacunin, or non-functional designated domains were generated and tested. These mutations inhibited OC cell growth, migration and invasion, and sensitised OC cells to cisplatin. Conversely, expression of WT ADAMTS16 promoted cisplatin resistance. In an orthotopic mouse model, mutated ADAMTS16 cells showed a better response to cisplatin, confirming the role of ADAMTS16 in chemotherapy resistance^[75]^. Therefore, as the overexpression of WT ADAMTS16 led to drug resistance, it would be interesting to further investigate the molecular mechanisms behind this. FN has recently been identified as an ADAMTS16 substrate, where ADAMTS16-mediated FN cleavage opposed FN fibrillogenesis^[8]^. Interestingly, ECM molecules have been implicated in etoposide resistance in retinoblastoma cell lines, where reduced FN expression, among others, was detected in resistant cells^[76]^. This raises the intriguing hypothesis that ADAMTS16 might regulate drug sensitivity through the modulation of FN abundance in the TME.

Other ADAMTS substrates were found associated with cancer development through bioinformatic analyses. ECM organisation and structural components such as collagen I, III, V and fibrillin-1 were found enriched and associated with ADAMTS12 expression in GC^[38]^. In addition, a cohort study which analysed 114 RCC patients samples collected from 1990 to 2005 from the Mayo Clinic Rochester has found that ADAMTS12 together with other ECM components, such as lumican and laminin-2, were upregulated in metastatic tumours compared to primary tumours^[77]^. Altogether, these pieces of evidence support the idea that AD-ATMSs might modulate tumour progression by regulating ECM cleavage in the TME. Given the importance of the ECM in cancer progression and its role in therapy resistance, it is fundamental to further characterise how different ADAMTSs impinge on ECM remodelling, as the TME and the ECM are currently being exploited as novel therapeutic targets in cancer.

### ADAMTSs in cancer-associated stromal cells

Cancer-associated fibroblasts play an important role in the TME. They interact with cancer cells and mostly favour tumour progression through secreting ECM components and modifying enzymes, including ADAMTSs. Importantly, normal fibroblasts can also be present in the TME and exert tumour suppressive functions^[78]^. For instance, even if ADAMTS12 has been found silenced in CRC cells, its expression was higher in stromal cells surrounding the tumour. Co-culture experiments using colon fibroblasts and CRC cell lines showed a strong induction in ADAMTS12 expression compared to fibroblasts alone, which was dependent on TGFβ signalling. Importantly, the co-culture with fibroblasts strongly impaired CRC cell growth and promoted apoptosis, suggesting that this might represent a protective response to counteract the epigenetic silencing of ADAMTS12 in CRC cells^[30]^. It is important to note that the direct involvement of ADAMTS12 in growth suppression, as well as the molecular mechanisms behind this, have not been elucidated. A more recent study also localised ADAMTS12 in fibroblasts and macrophages located around CRC cells at the invasive margins and reported that low levels of ADAMTS12 in the stroma resulted in poor overall survival of CRC patients, supporting the hypothesis that stromal ADAMTS12 opposes tumour progression^[79]^. It is tempting to speculate that upregulation of ADAMTS12 in fibroblasts could be induced by cancer cells during the early stages of tumour development, while at later stages more aggressive cancer cells prevent stromal accumulation of ADAMTS12. It would be interesting to determine how cancer cells affect protease stromal expression, as well as the molecular mechanisms through which ADAMTS12 opposes cell growth.

In BC, ADAMTS3 has been shown to be downregulated in myoepithelial cells at the ductal carcinoma in situ stage. As in this context ADAMTS3 directly cleaves FN, the loss of ADAMTS3 expression results in FN accumulation (Fig. 4C), which promotes cancer cell migration via the interaction with α5 integrin, resulting in the transition to invasive carcinoma^[80]^. Altogether, these highlight the multiple roles that ADAMTS family members play in the tumour microenvironment, in addition to directly affecting cancer cell behaviours.

### ADAMTSs regulate cell-ECM interaction in cancer

Cells interact with the ECM through a variety of plasma membrane receptors, including integrins and proteoglycans of the syndecan family. In OC, treatment with conditioned media from either ADAMTS1 WT or a catalytically inactive (CI) mutant inhibited fibroblast migration. This suggests that the enzymatic activity of ADAMTS1 is not required for its anti-migratory properties. Consistently, ADAMTS1 KO cells showed increased cell migration, accompanied by an increase in adhesion signalling mediated by Src and focal adhesion kinase (FAK). In addition, ADAMTS1 deletion promoted the polarised activation of Cdc42 at the leading edge^[81]^, suggesting that secreted ADAMTS1 might control cell migration through the modulation of adhesion signalling and cell polarity. It is possible that this effect is mediated by syndecan-4, as this plasma membrane proteoglycan has been reported to be cleaved by ADAMTS1, resulting in syndecan-4 ectodomain shedding^[82]^. Syndecan-4 is an integrin co-receptor, which promotes adhesion signalling, small GTPase activation at the leading edge and cell migration. Therefore, it would be interesting to test whether ADAMTS1 KO-driven cell migration requires syndecan-4 function.

Similarly, the overexpression of either the full length ADAMTS15 or a CI mutant decreased BC cell adhesion and migration on FN or laminin, but not on collagen I. This indicates that ADAMTS15 suppresses BC cell migration independently of its aggrecanase activity, but in an ECM-dependent manner^[83]^. Mechanistically, ADAMTS15 WT or CI overexpression increased the surface levels of syndecan-4 and syndecan-4 knockdown opposed ADAMTS15-driven inhibition of BC cell migration. As ADAMTS15 has a much weaker enzymatic activity compared to other ADAMTSs, it is intriguing to hypothesise that the pericellular accumulation of ADAMTS15 could prevent ADAMTS1-dependent syndecan-4 cleavage and shedding, therefore resulting in its accumulation at the plasma membrane. It has been reported that either excessive accumulation or complete loss on syndecan-4 impairs cell migration^[84]^. Therefore, the fine balance between ADAMTS1 and ADAMTS15 could represent a mechanism to precisely tune the levels of syndecan-4 at the plasma membrane, to drive cancer cell migration (Fig. 4D). Additionally, tail vein injections of ADAMTS15 WT- and CI-expressing cells led to the formation of fewer liver micrometastases, compared to control cells, consistent with the ADAMTS15 anti-migratory effects observed *in vitro*. Surprisingly, the overexpression of ADAMTS15 WT, but not CI, promoted lung metastasis formation, indicating opposite roles of ADAMTS15 in the regulation of metastasis formation *in vivo*, as well as differential requirements of ADAMTS15 catalytic activity depending on the metastatic site^[83]^. This could be driven by specific ECM signatures at different metastatic sites and further work is required to elucidate the molecular mechanisms through which ADAMTS15 controls metastasis formation.

### ADAMTSs regulate cancer progression through interactions with multiple signalling pathways

Epithelial-to-mesenchymal transition (EMT) is a process involving loss of epithelial features, such as cell-cell and cell-ECM adhesion, E-cadherin and β_4_ integrin expression, and gain of mesenchymal properties, including the expression of vimentin, FN, β_1_ and β_3_ integrins^[85]^. Core EMT transcription factors modulate the acquisition of the mesenchymal properties that cancer cells gain during EMT, leading to the activation of signalling pathways that will maintain the phenotype^[86]^. Over-activation of signalling pathways such as Wnt/β-catenin, TGFβ, mitogen-activated protein kinase kinase (MEK)/extracellular signal-regulated kinase (ERK), phosphoinositide 3-kinase/protein kinase B (PI3K/AKT) and nuclear factor kappa B (NF-κB) has been widely reported to contribute to cancer cell survival and the acquisition of a meta-static phenotype^[87]^. Interestingly, ADAMTS family members have been found to associate with cytokines and growth factors^[88]^, thereby modulating downstream signalling pathways.

### ADAMTSs regulate EMT to promote invasion and metastasis

**Fig. 5:**
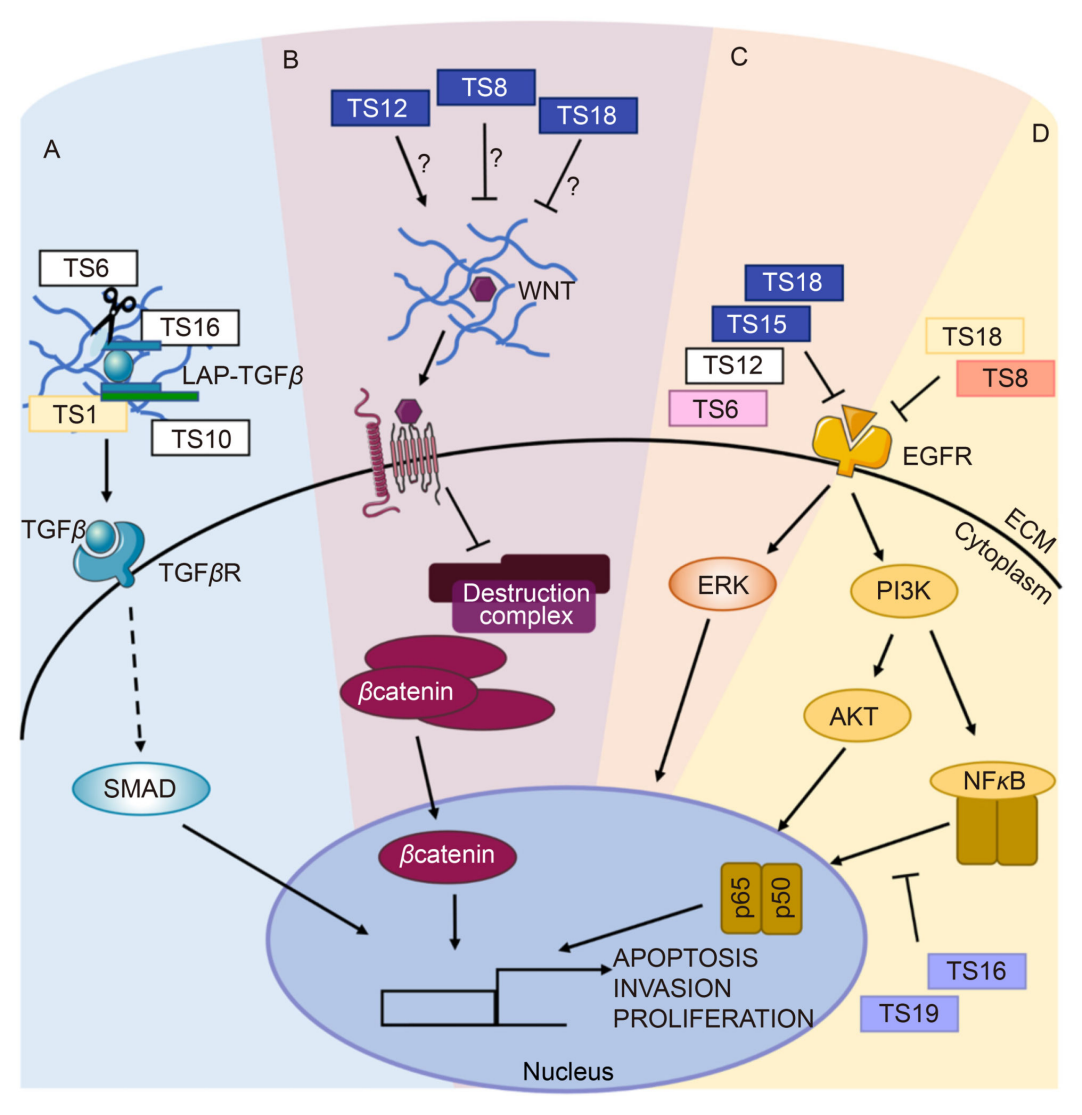
ADAMTSs crosstalk with signalling pathways regulates cancer progression. A. ADAMTS1, 6, 10 and 16 can activate TGFβ by cleaving the ECM-bound LAP domain, leading to promotion of TGFβ signalling that drives EMT and cell migration. B. In CRC, ADAMTS12 activates the Wnt signalling pathway while ADAMTS8 and 18 inhibit Wnt activation, potentially by stabilising Wnt in the ECM. C. ADAMTS6, 12, 15 and 18 inhibit ERK signalling potentially by regulating EGFR activation, in different cancer types. D. AKT activation is negatively regulated by ADAMTS8 and 18, in oesophageal cancer and LC respectively. NFκB is negatively regulated by ADAMTS16 and 19 in GC, leading to decreased invasion and proliferation of cancer cells. ADAMTSs have been colour coded based on cancer type, light yellow for LC; dark blue for CRC; pink for BC; salmon for oesophageal cancer and purple for GC. Image generated with elements from Servier Medical Art, licensed under CC BY 4.0.

ADAMTSs were found to contribute to EMT, in a context-dependent manner. ADAMTS1 was found to be upregulated in LC and to promote EMT in non-small cell LC cells, both *in vitro* and *in vivo*. Overexpression of ADAMTS1 resulted in increased migration and invasion, coupled with downregulation of E-cadherin and upregulation of the mesenchymal markers N-cadherin and vimentin. Conversely, ADAMTS1 knock-down impaired EMT and cell migration. Moreover, the subcutaneous injection of ADAMTS1 overexpressing cells in nude mice resulted in more lung metastasis compared to control cells. Mechanistically, ADAMTS1 promoted EMT through the activation of TGFβ signalling (Fig. 5A), as TGFβ pharmacological inhibition or siRNA-mediated downregulation opposed ADAMTS1 driven cell migration^[89]^. TGFβ is synthesised as a dimer and is maintained inactive by the latency-associated peptide-TGFβ (LAP-TGFβ). There are two main TGFβ activation mechanisms, either via RGD motif-containing integrins binding to LAP or via the cleavage of LAP by ECM proteases^[90]^. Indeed, ADAMTS1 was previously shown to directly interact with LAP-TGFβ, to promote TGFβ activation^[91]^, in a catalytic activity-independent manner. Similarly, ADAMTS16 was shown to interact with and activate LAP-TGFβ in cardiac fibroblasts^[92]^. Other members of the ADAMTS family, including ADAMTS6 and ADAMTS10, have been shown to bind to ECM proteins that regulate the extracellular availability of TGFβ (Fig. 5A). In chondrocyte, ADAMTS6 was found to increase TGFβ activation in a dose-dependent manner, through a mechanism predominantly mediated by its catalytic activity, although some activation could also be observed by a catalytically inactive mutant. ADAMTS10 was also able to induce TGFβ activation, but this was to a much lower extent than ADAMTS6. In addition, both ADAMTS6 and ADAMTS10 could also indirectly activate TGFβ by increasing cellular tension^[93]^. These data indicate that the ability to promote TGFβ signalling is shared between different members of the ADAMTS family, suggesting that they could play a role in controlling EMT in cancer. Indeed, in CRC, ADAMTS6 promotes EMT to drive cancer cell migration and invasion^[94]^. Therefore, ADAMTSs could represent promising therapeutic targets to prevent TGFβ-driven EMT.

Bioinformatic analysis in PDAC cells showed positive correlation between ADAMTS12, EMT markers and EMT transcription factors^[95]^. In addition, ADAMTS12 mRNA was found to be upregulated in the stroma of PDAC patients, compared to non-tumoral fibrotic tissue, among other genes linked to ECM remodelling, cell migration and cell cycle^[96]^. ADAMTS12 down regulation in PDAC cells significantly reduced migration in transwell chambers and wound healing assays, while tail vein injections of ADAMTS12 KO cells resulted in a reduction of lung metastasis formation, indicating that ADAMS12 is required for PDAC cell migration *in vitro* and *in vivo*. Mechanistically, ADAMTS12 downregulation resulted in elevated E-cadherin levels and a concomitant reduction in N-cadherin and vimentin, suggesting that ADAMTS12 might regulate EMT^[95]^. The molecular mechanisms underpinning this have not been elucidated yet. TGFβ has been identified as an ADAMTS12 interactor in a mass spectrometry screen^[97]^, suggesting a similar mechanism to ADAMTS1 and 6 in controlling TGFβ availability. Alternatively, as ADAMTS12 has been linked to the activation of Wnt/β-catenin signalling (see below) and Wnt/β-catenin is critical in EMT regulation^[98]^, it is possible to speculate that ADAMTS12 might control EMT through the regulation of Wnt signalling. Conversely, ADAMTS12 was found to inhibit Hepatocyte growth factor (HGF)-induced EMT in Madin-Darby Canine Kidney (MCDK) cells, and mice injected with lung adenocarcinoma A549 cells overexpressing ADAMTS12 showed a significantly reduced tumour volume and size^[57]^, indicating that ADAMTS12-dependent regulation of EMT might be tissue specific.

### ADAMTSs crosstalk with the Wnt/β-catenin pathway

Wnt/β-catenin deregulation is associated with cancer progression, by facilitating stem cell renewal, differentiation and proliferation^[99]^. Wnt canonical pathway is activated by the binding of Wnt to Frizzled, which phosphorylates Dishevelled, leading to the activation of co-receptor LRP5/6. This complex stabilises β-catenin by blocking its degradation. The accumulation of β-catenin in the nucleus then activates Wnt downstream genes^[100]^. ADAMTS12 is upregulated in CRC and high ADAMTS12 levels correlate with poor prognosis. *In vitro*, ADAMTS12 overexpression promoted cell proliferation and migration, while ADAMTS12 downregulation impaired both processes. Overexpression of ADAMTS12 in HEK239 cells stimulated the transcriptional activity of Wnt/β-catenin, while ADAMTS12 knockdown in CRC cells decreased the expression of the Wnt/β-catenin target genes myc and cyclin D1, suggesting that ADAMTS12 might control CRC progression through the modulation of the Wnt/ β-catenin pathway (Fig. 5B), which is a key driver of CRC^[101]^. Contrarily, ADAMTS8 acts as a tumour suppressor in CRC by inactivating the Wnt pathway. Overexpression of ADAMTS8 was shown to reduce β-catenin levels, as well as the phosphorylation of GSK3β, leading to reduced CRC cell invasion and migration *in vitro* and reduced tumour growth *in vivo* (Fig. 5B)^[23]^. Similarly, ADAMTS18 deficiency in CRC was associated with increased β-catenin nuclear translocation, suggesting Wnt activation^[102]^. The molecular mechanisms through which ADAMTSs control Wnt signalling have not been elucidated yet. Interestingly, Wnt has been shown to be stabilised in the extracellular space via the interaction with proteoglycans in the ECM^[103]^. Indeed, proteoglycans were shown to be required to sustain Wnt signalling, by modulating Wnt availability. It is therefore tempting to speculate that, by remodelling the ECM, ADAMTSs might impinge on Wnt mobility in the extracellular space, therefore impacting Wnt signalling (Fig. 5B).

### ADAMTSs might control PI3K/AKT and ERK signalling through interaction with EGFR

Epidermal growth factor receptor (EGFR) was found hyper-activated in multiple cancer types, resulting in cancer cell growth, survival, metastasis and therapy resistance. EGFR is found at the plasma membrane as an inactive monomer. Upon ligand binding, a conformational change leads to the formation of homo- or heterodimers, promoting the activation of downstream signalling pathways such as PI3K/AKT and MARK/ERK^[104]^. PI3K/AKT is one of the most frequently over-activated pathways in cancer, contributing to all hallmarks of cancer. ERK is phosphorylated via the activation of the Ras/Raf/MAPK signalling cascade leading to activation of downstream factors that contribute to cancer progression, like c-myc and c-Fos^[105,106]^.

In LC cells, ADAMTS18 overexpression decreased EGFR/AKT activation^[29]^, while secreted ADAMTS8 was found to reduce EGFR/MAPK/ERK signalling in oesophageal cancer cells^[22]^. In BC cell lines, overexpression of ADAMTS6 reduced invasion and migration, coupled with decreased EGFR and ERK activation^[36]^. Similarly, ADAMTS12 and ADAMTS15 overexpression resulted in reduced phospho-ERK levels, while ADAMTS15 silencing led to the overactivation of the ERK signalling pathway^[39,57]^. In addition, ADAMTS18 KO in a CRC model resulted in increased ERK and p38 MAPK activation^[102]^. This suggests that several ADAMTS family members might be negative regulators of AKT and ERK signalling, potentially through the regulation of EGFR activation (Fig. 5C and D). Cleaved fragments of ADAMTS1, which contain at least one TSR-1 motif, have been shown to prevent EGFR and ERK activation by negatively modulating the bioavailability and activity of the heparin-binding EGF^[107]^. This suggests that TSR-1 motifs from ADAMTS family members could control EGFR activation via a similar mechanism. Indeed, multiple heparin binding sequences have been identified in the TSR-1 domain of ADAMTSs^[16]^. Furthermore, the binding of ADAMTS6^[108]^, 8 ^[109]^ and 15 ^[110]^ to heparin has been previously observed. Further work will be needed to fully elucidate the role of ADAMTSs in controlling EGF bioavailability and downstream signalling in different cancer types and to establish whether this could lead to novel therapeutic strategies to prevent EGFR-driven tumorigenesis. Alternatively, ADAMTSs might regulate PI3K/AKT and ERK signalling through ECM remodelling, as several ECM components have been implicated in the modulation of these pathways^[1]^. Indeed, ADAMTS18 deficiency resulted in the accumulation of several ECM components, including FN, in mouse mammary tumours and potentiated the activation of ERK and AKT^[111]^. As ADAMTS18 has been shown to cleave FN^[9]^, this ECM component accumulates in ADAMTS18 KO tumours, resulting in integrin dependent ERK and AKT activation. Further work is needed to fully elucidate the crosstalk between ADAMTS-driven ECM remodelling and integrin activation.

In GC cells, ADAMTS19 has been found to be downregulated and it has been suggested to interact with the NFκB subunit p65, preventing p65 nuclear localisation and NFκB activation^[112]^. Conversely, ADAMTS16 was found to be upregulated in GC and to promote NFκB signalling by stimulating NFκB nuclear translocation, potentially by interacting with the NFκB regulator IκBα (Fig. 5D)^[113]^. These interactions are supposed to happen within the cells and a potential mechanism through which secreted ADAMTS19 and 16 are transported to the cytosol is still lacking. Interestingly, some ADAMTS family members have been shown to be localised in the nucleus. Indeed, ADAMTS4 and the ADAMTS5 fragment TS5-p45 accumulated in the nucleus of smooth muscle cells and endothelial cells, respectively, driving apoptosis^[114,115]^; while ADAMTS1, but not ADAMTS4 and 5, localised to the nucleus of non-transformed mammary epithelial cells and BC cells^[116]^. This raised the intriguing possibility that ADAMTSs might regulate the activity of transcription factors, including p65, in the nucleus. Alternatively, it is possible that ADAMTS-dependent regulation of NFκB might be indirect, as NFκB can be activated downstream of AKT^[117]^. Indeed, in RCC, ADAMTS18 downregulation impaired both AKT and NFκB activation^[118]^. Further work is needed to deeply characterise any intracellular role of ADAMTS family members and how this impacts on intracellular signalling pathways, as opposed to the extracellular modulation of growth factor availability.

### Conclusions and future research

In this review, we have highlighted the impact of ADAMTS dysregulation on the progression of multiple cancer types. ADAMTSs can promote or suppress cancer cell invasion and metastasis by promoting ECM remodelling and/or regulating intracellular signalling pathways. ADAMTS function has been investigated thoroughly in some cancer types, like LC, BC, CRC, and GC. Notwithstanding, in other cancer types such as RCC, PC and OC, ADAMTSs were found dysregulated but further research needs to be carried out to determine their impact on tumorigenesis. Since several *in vivo* studies rely on the injection of human cells in immunocompromised mice, it would be important to further these with the use of genetic mouse models, whether the TME could be more clinically relevant. In addition, the use to organoid cultures and patient-derived xenograft will be beneficial to further elucidate the effect of ADAMTS inhibition on tumour progression. More complex co-culture models would be essential to characterise how ADATMS activity impinges on the interaction between different TME components at the molecular level. Within the TME, ADAMTSs can be secreted by cancer cells or by cancer associated stromal cells, leading to both pro- and anti-tumorigenic environments. This has been found to depend on the cancer type and the ADAMTS family members, and it could potentially be due to the presence of different ECM components in the TME, which can be cleaved by different ADAMTSs. It is becoming clearer that ADAMTSs also control tumorigenesis through direct interaction and/or cleavage of growth factors, thereby modulating downstream signalling pathways, including TGFβ, EGFR/ERK and NF-κB. As these functions have been mainly detected in cancer, this opens the possibility of targeting ADAMTSs for future therapeutic approaches. Indeed, small molecule inhibitors targeting aggrecanases, including ADAMTS4 and 5, are in phase III clinical trials for osteoarthritis treatment^[119]^. A challenge of targeting ADAMTSs for cancer therapy will be the identification of the ECM and/or growth factor interactions that promote cancer versus the ones that suppress the disease and the development of high specificity inhibitors, to be able to specifically block the tumour-promoting activity without impacting on any potential tumour suppressive roles.

## Abbreviations

ADAM: A Disintegrin and Metalloproteinase
ADAMTS: ADAMs with thrombospondin(-like) motifs
ADC: 5-aza-2 ′ - deoxycytidine
BC: breast cancer
BPH: benign prostatic hyperplasia
CAF: cancer associated fibroblast
CI: catalytically inactive
CRC: colorectal cancer
DNMT: DNA methyltransferase
ECM: extracellular matrix
EGFR: epidermal growth factor receptor
EMT: epithelial-to-mesenchymal transition
FN: fibronectin
GC: gastric cancer
LAP-TGFβ: latency-associated peptide-TGFβ
LC: lung cancer
MMP: matrix-metalloproteinase
OC: ovarian cancer
PC: prostate cancer
PDAC: pancreatic ductal adenocarcinoma
RCC: renal cell carcinoma
RNF180: ring finger protein 180
TGFβ: transforming growth factor beta
TIMPS: tissue inhibitors of metalloproteinases
TME: tumour microenvironment
TSR-1: thrombospondin type 1-like motif

## References

[1] Pickup MW, Mouw JK, Weaver VM (2014). The extracellular matrix modulates the hallmarks of cancer. EMBO Rep.

[2] Quesnel A, Broughton A, Karagiannis GS (2022). Message in the bottle: regulation of the tumor microenvironment via exosome-driven proteolysis. Cancer Metastasis Rev.

[3] Niland S, Eble JA (2020). Hold on or Cut? Integrin- and MMP-Mediated Cell-Matrix Interactions in the Tumor Microenvironment. Int J Mol Sci.

[4] Costa S, Ragusa MA, Lo Buglio G (2022). The Repertoire of Tissue Inhibitors of Metalloproteases: Evolution, Regulation of Extracellular Matrix Proteolysis, Engineering and Therapeutic Challenges. Life (Basel).

[5] Kuno K, Kanada N, Nakashima E (1997). Molecular cloning of a gene encoding a new type of metalloproteinase-disintegrin family protein with thrombospondin motifs as an inflammation associated gene. J Biol Chem.

[6] Stanton H, Melrose J, Little CB (2011). Proteoglycan degradation by the ADAMTS family of proteinases. Biochim Biophys Acta.

[7] Kelwick R, Desanlis I, Wheeler GN (2015). The ADAMTS (A Disintegrin and Metalloproteinase with Thrombospondin motifs) family. Genome Biol.

[8] Schnellmann R, Sack R, Hess D (2018). A Selective Extracellular Matrix Proteomics Approach Identifies Fibronectin Proteolysis by A Disintegrin-like and Metalloprotease Domain with Thrombospondin Type 1 Motifs (ADAMTS16) and Its Impact on Spheroid Morphogenesis. Mol Cell Proteomics.

[9] Ataca D, Aouad P, Constantin C (2020). The secreted protease Adamts18 links hormone action to activation of the mammary stem cell niche. Nat Commun.

[10] Blobel CP, Apte S (2022). ADAMs and ADAMTSs. Encyclopedia Resp Med.

[11] Nandadasa S, Nelson CM, Apte SS (2015). ADAMTS9-Mediated Extracellular Matrix Dynamics Regulates Umbilical Cord Vascular Smooth Muscle Differentiation and Rotation. Cell Rep.

[12] McCulloch DR, Nelson CM, Dixon LJ (2009). ADAMTS metalloproteases generate active versican fragments that regulate interdigital web regression. Dev Cell.

[13] Dupuis LE, McCulloch DR, McGarity JD (2011). Altered versican cleavage in ADAMTS5 deficient mice; a novel etiology of myxomatous valve disease. Dev Biol.

[14] Silver DL, Hou L, Somerville R (2008). The secreted metalloprotease ADAMTS20 is required for melanoblast survival. PLoS Genet.

[15] Shindo T, Kurihara H, Kuno K (2000). ADAMTS-1: a metalloproteinase-disintegrin essential for normal growth, fertility, and organ morphology and function. J Clin Invest.

[16] Jones GC, Riley GP (2005). ADAMTS proteinases: a multi-domain, multi-functional family with roles in extracellular matrix turnover and arthritis. Arthritis Res Ther.

[17] Li T (2022). The Mechanism and Role of ADAMTS Protein Family in Osteoarthritis. Biomolecules.

[18] Bartha Á, Győrffy B (2021). TNMplot. com: A Web Tool for the Comparison of Gene Expression in Normal, Tumor and Metastatic Tissues. Int J Mol Sci.

[19] Chen J, Zhang J, Li X (2016). Downregulation of ADAMTS8 by DNA Hypermethylation in Gastric Cancer and Its Clinical Significance. Biomed Res Int.

[20] Sun W, Ma G, Zhang L (2021). DNMT3A-mediated silence in ADAMTS9 expression is restored by RNF180 to inhibit viability and motility in gastric cancer cells. Cell Death Dis.

[21] Zhang C, Shao Y, Zhang W (2010). High-resolution melting analysis of ADAMTS9 methylation levels in gastric, colorectal, and pancreatic cancers. Cancer Genet Cytogenet.

[22] Choi GC, Li J, Wang Y (2014). The metalloprotease ADAMTS8 displays antitumor properties through antagonizing EGFR-MEK-ERK signaling and is silenced in carcinomas by CpG methylation. Mol Cancer Res.

[23] Li L, Yuan S, Zhao X (2020). ADAMTS8 is frequently down-regulated in colorectal cancer and functions as a tumor suppressor. Biochem Biophys Res Commun.

[24] Chen L, Tang J, Feng Y (2017). ADAMTS9 is Silenced by Epigenetic Disruption in Colorectal Cancer and Inhibits Cell Growth and Metastasis by Regulating Akt/p53 Signaling. Cell Physiol Biochem.

[25] Kordowski F, Kolarova J, Schafmayer C (2018). Aberrant DNA methylation of ADAMTS16 in colorectal and other epithelial cancers. BMC Cancer.

[26] Alonso S, González B, Ruiz-Larroya T (2015). Epigenetic inactivation of the extracellular matrix metallopeptidase ADAMTS19 gene and the metastatic spread in colorectal cancer. Clin Epigenetics.

[27] Xu B, Zhang L, Luo C (2015). Hypermethylation of the 16q23.1 tumor suppressor gene ADAMTS18 in clear cell renal cell carcinoma. Int J Mol Sci.

[28] Daniunaite K, Bakavicius A, Zukauskaite K (2021). Promoter methylation of PRKCB, ADAMTS12, and NAALAD2 is specific to prostate cancer and predicts biochemical disease recurrence. Int J Mol Sci.

[29] Zhang Y, Xu H, Mu J (2019). Inactivation of ADAMTS18 by aberrant promoter hypermethylation contribute to lung cancer progression. J Cell Physiol.

[30] Moncada-Pazos A, Obaya AJ, Fraga MF (2009). The ADAMTS12 metalloprotease gene is epigenetically silenced in tumor cells and transcriptionally activated in the stroma during progression of colon cancer. J Cell Sci.

[31] Nepali K, Liou JP (2021). Recent developments in epigenetic cancer therapeutics: clinical advancement and emerging trends. J Biomed Sci.

[32] Lee HC, Chang CY, Wu KL (2022). The Therapeutic Potential of ADAMTS8 in Lung Adenocarcinoma without Targetable Therapy. J Pers Med.

[33] Catalanotto C, Cogoni C, Zardo G (2016). MicroRNA in Control of Gene Expression: An Overview of Nuclear Functions. Int J Mol Sci.

[34] Li M, Liu L, Zang W (2015). miR-365 overexpression promotes cell proliferation and invasion by targeting ADAMTS-1 in breast cancer. Int J Oncol.

[35] Liu F, Zhuang L, Wu R (2019). miR-365 inhibits cell invasion and migration of triple negative breast cancer through ADAM10. J BUON.

[36] Xie Y, Gou Q, Xie K (2016). ADAMTS6 suppresses tumor progression via the ERK signaling pathway and serves as a prognostic marker in human breast cancer. Oncotarget.

[37] Porter S, Span PN, Sweep FC (2006). ADAMTS8 and ADAMTS15 expression predicts survival in human breast carcinoma. Int J Cancer.

[38] Liang L, Zhu JH, Chen G (2020). Prognostic Values for the mRNA Expression of the ADAMTS Family of Genes in Gastric Cancer. J Oncol.

[39] Viloria CG, Obaya AJ, Moncada-Pazos A (2009). Genetic inactivation of ADAMTS15 metalloprotease in human colorectal cancer. Cancer Res.

[40] Lima MA, Dos Santos L, Turri JA (2016). Prognostic Value of ADAMTS Proteases and Their Substrates in Epithelial Ovarian Cancer. Pathobiology.

[41] Haraguchi N, Ohara N, Koseki J (2017). High expression of ADAMTS5 is a potent marker for lymphatic invasion and lymph node metastasis in colorectal cancer. Mol Clin Oncol.

[42] Cross NA, Chandrasekharan S, Jokonya N (2005). The expression and regulation of ADAMTS-1, -4, -5, -9, and -15, and TIMP-3 by TGFbeta1 in prostate cells: relevance to the accumulation of versican. Prostate.

[43] Mohammed MA, Seleim MF, Abdalla MS (2013). Urinary high molecular weight matrix metalloproteinases as non-invasive biomarker for detection of bladder cancer. BMC Urol.

[44] Zhang L, Liu Y, Zheng P (2018). Downregulation of ADAMTS18 May Serve as a Poor Prognostic Biomarker for Cervical Cancer Patients. Appl Immunohistochem Mol Morphol.

[45] Zhou D, Tang W, Liu X (2017). Clinical verification of plasma messenger RNA as novel noninvasive biomarker identified through bioinformatics analysis for lung cancer. Oncotarget.

[46] Shen Q, Polom K, Williams C (2019). A targeted proteomics approach reveals a serum protein signature as diagnostic biomarker for resectable gastric cancer. EBioMedicine.

[47] Wang B, Chen S, Zhao JQ (2019). ADAMTS-1 inhibits angiogenesis via the PI3K/Akt-eNOS-VEGF pathway in lung cancer cells. Transl Cancer Res.

[48] Lee HC, Chang CY, Huang YC (2022). Downregulated ADAMTS1 Incorporating A2M Contributes to Tumorigenesis and Alters Tumor Immune Microenvironment in Lung Adenocarcinoma. Biology (Basel).

[49] Guo R, Yang J, Liu X (2018). Increased von Willebrand factor over decreased ADAMTS-13 activity is associated with poor prognosis in patients with advanced non-small-cell lung cancer. J Clin Lab Anal.

[50] Liu X, Chen X, Yang J (2017). Association of ABO blood groups with von Willebrand factor, factor VIII and ADAMTS-13 in patients with lung cancer. Oncol Lett.

[51] Zhang Y, Hu K, Qu Z (2022). ADAMTS8 inhibited lung cancer progression through suppressing VEGFA. Biochem Biophys Res Commun.

[52] Freitas VM, do Amaral JB, Silva TA (2013). Decreased expression of ADAMTS-1 in human breast tumors stimulates migration and invasion. Mol Cancer.

[53] Porter S, Scott SD, Sassoon EM (2004). Dysregulated expression of adamalysin-thrombospondin genes in human breast carcinoma. Clin Cancer Res.

[54] Xu H, Xiao Q, Fan Y (2017). Epigenetic silencing of ADAMTS18 promotes cell migration and invasion of breast cancer through AKT and NF-κB signaling. Cancer Med.

[55] Chen J, Luo Y, Zhou Y (2018). Promotion of Tumor Growth by ADAMTS4 in Colorectal Cancer: Focused on Macrophages. Cell Physiol Biochem.

[56] Song C, Chen J, Zhang C (2022). An Integrated Pan-Cancer Analysis of ADAMTS12 and Its Potential Implications in Pancreatic Adenocarcinoma. Front Oncol.

[57] Llamazares M, Obaya AJ, Moncada-Pazos A (2007). The ADAMTS12 metalloproteinase exhibits anti-tumorigenic properties through modulation of the Ras-dependent ERK signalling pathway. J Cell Sci.

[58] Walker C, Mojares E, del Río Hernández A (2018). Role of Extracellular Matrix in Development and Cancer Progression. Int J Mol Sci.

[59] Yue B (2014). Biology of the extracellular matrix: an overview. J Glaucoma.

[60] Shao X, Gomez CD, Kapoor N (2023). MatrisomeDB 2.0: 2023 updates to the ECM-protein knowledge database. Nucleic Acids Res.

[61] Steeg PS (2006). Tumor metastasis: mechanistic insights and clinical challenges. Nat Med.

[62] Maquart FX, Pasco S, Ramont L (2004). An introduction to matrikines: extracellular matrix-derived peptides which regulate cell activity. Implication in tumor invasion. Crit Rev Oncol Hematol.

[63] Rose KWJ, Taye N, Karoulias SZ (2021). Regulation of ADAMTS Proteases. Front Mol Biosci.

[64] Ricciardelli C, Russell DL, Ween MP (2007). Formation of hyaluronan- and versican-rich pericellular matrix by prostate cancer cells promotes cell motility. J Biol Chem.

[65] Sakko AJ, Ricciardelli C, Mayne K (2003). Modulation of prostate cancer cell attachment to matrix by versican. Cancer Res.

[66] Mead TJ, Nelson CM (2018). ADAMTS9-Regulated Pericellular Matrix Dynamics Governs Focal Adhesion-Dependent Smooth Muscle Differentiation. Cell Rep.

[67] Wight TN, Kang I, Evanko SP (2020). Versican-A Critical Extracellular Matrix Regulator of Immunity and Inflammation. Front Immunol.

[68] Ghosh S, Albitar L, LeBaron R (2010). Up-regulation of stromal versican expression in advanced stage serous ovarian cancer. Gynecol Oncol.

[69] Papadas A, Arauz G, Cicala A (2020). Versican and Versican-matrikines in Cancer Progression, Inflammation, and Immunity. J Histochem Cytochem.

[70] Papadas A, Deb G, Cicala A (2022). Stromal remodeling regulates dendritic cell abundance and activity in the tumor microenvironment. Cell Rep.

[71] Fontanil T, Álvarez-Teijeiro S, Villaronga MÁ (2017). Cleavage of Fibulin-2 by the aggrecanases ADAMTS-4 and ADAMTS-5 contributes to the tumorigenic potential of breast cancer cells. Oncotarget.

[72] Fontanil T, Rúa S, Llamazares M (2014). Interaction between the ADAMTS-12 metalloprotease and fibulin-2 induces tumor-suppressive effects in breast cancer cells. Oncotarget.

[73] Kischel P, Waltregny D, Dumont B (2010). Versican overexpression in human breast cancer lesions: known and new isoforms for stromal tumor targeting. Int J Cancer.

[74] Liu Y, Yasukawa M, Chen K (2015). Association of Somatic Mutations of ADAMTS Genes With Chemotherapy Sensitivity and Survival in High-Grade Serous Ovarian Carcinoma. JAMA Oncol.

[75] Yasukawa M, Liu Y, Hu L (2017). ADAMTS16 mutations sensitize ovarian cancer cells to platinum-based chemotherapy. Oncotarget.

[76] Reinhard J, Wagner N, Krämer MM (2020). Expression Changes and Impact of the Extracellular Matrix on Etoposide Resistant Human Retinoblastoma Cell Lines. Int J Mol Sci.

[77] Ho TH, Serie DJ, Parasramka M (2017). Differential gene expression profiling of matched primary renal cell carcinoma and metastases reveals upregulation of extracellular matrix genes. Ann Oncol.

[78] Alkasalias T, Flaberg E, Kashuba V (2014). Inhibition of tumor cell proliferation and motility by fibroblasts is both contact and soluble factor dependent. Proc Natl Acad Sci U S A.

[79] Wang D, Zhu T, Zhang FB (2011). Expression of ADAMTS12 in colorectal cancer-associated stroma prevents cancer development and is a good prognostic indicator of colorectal cancer. Dig Dis Sci.

[80] Gibson SV, Madzharova E, Tan AC (2023). ADAMTS3 restricts cancer invasion in models of early breast cancer progression through enhanced fibronectin degradation. Matrix Biol.

[81] de Assis Lima M, da Silva SV, Serrano-Garrido O (2021). Metalloprotease ADAMTS-1 decreases cell migration and invasion modulating the spatiotemporal dynamics of Cdc42 activity. Cell Signal.

[82] Rodríguez-Manzaneque JC, Carpizo D, del Carmen Plaza-Calonge M (2009). Cleavage of syndecan-4 by ADAMTS1 provokes defects in adhesion. Int J Biochem Cell Biol.

[83] Kelwick R, Wagstaff L, Decock J (2015). Metalloproteinase-dependent and -independent processes contribute to inhibition of breast cancer cell migration, angiogenesis and liver metastasis by a disintegrin and metalloproteinase with thrombospondin motifs-15. Int J Cancer.

[84] Wilcox-Adelman SA, Denhez F, Iwabuchi T (2002). Syndecan-4: dispensable or indispensable?. Glycoconj J.

[85] Dongre A, Weinberg RA (2019). New insights into the mechanisms of epithelial-mesenchymal transition and implications for cancer. Nat Rev Mol Cell Biol.

[86] Li D, Xia L, Huang P (2023). Heterogeneity and plasticity of epithelial-mesenchymal transition (EMT) in cancer metastasis: Focusing on partial EMT and regulatory mechanisms. Cell Prolif.

[87] Yip HYK, Papa A (2021). Signaling Pathways in Cancer: Therapeutic Targets, Combinatorial Treatments, and New Developments. Cells.

[88] Yang H, Khalil RA (2022). ADAM and ADAMTS disintegrin and metalloproteinases as major factors and molecular targets in vascular malfunction and disease. Adv Pharmacol.

[89] Hu X, Jiang C, Hu N (2023). ADAMTS1 induces epithelial-mesenchymal transition pathway in non-small cell lung cancer by regulating TGF-β. Aging (Albany NY).

[90] Tzavlaki K, Moustakas A (2020). TGF-β Signaling. Biomolecules.

[91] Bourd-Boittin K, Bonnier D, Leyme A (2011). Protease profiling of liver fibrosis reveals the ADAM metallopeptidase with thrombospondin type 1 motif, 1 as a central activator of transforming growth factor beta. Hepatology.

[92] Yao Y, Hu C, Song Q (2020). ADAMTS16 activates latent TGF-β, accentuating fibrosis and dysfunction of the pressure-overloaded heart. Cardiovasc Res.

[93] Cain SA, Woods S, Singh M (2022). ADAMTS6 cleaves the large latent TGFβ complex and increases the mechanotension of cells to activate TGFβ. Matrix Biol.

[94] Wang YP, Zhao YJ, Kong XL (2020). A metalloproteinase of the disintegrin and metalloproteinases and the ThromboSpondin Motifs 6 as a novel marker for colon cancer: functional experiments. Genet Mol Biol.

[95] He RZ, Zheng JH, Yao HF (2023). ADAMTS12 promotes migration and epithelial-mesenchymal transition and predicts poor prognosis for pancreatic cancer. Hepatobiliary Pancreat Dis Int.

[96] Robin F, Angenard G, Cano L (2020). Molecular profiling of stroma highlights stratifin as a novel biomarker of poor prognosis in pancreatic ductal adenocarcinoma. Br J Cancer.

[97] Zou R, Gu R, Tu X (2023). Effects of metalloprotease ADAMTS12 on cervical cancer cell phenotype and its potential mechanism. Discov Oncol.

[98] Basu S, Cheriyamundath S, Ben-Ze’ev A (2018). Cell-cell adhesion: linking Wnt/β-catenin signaling with partial EMT and stemness traits in tumorigenesis. F1000Res.

[99] Zhang Y, Wang X (2020). Targeting the Wnt/β-catenin signaling pathway in cancer. J Hematol Oncol.

[100] Komiya Y, Habas R (2008). Wnt signal transduction pathways. Organogenesis.

[101] Li C, Luo X, Huang B (2020). ADAMTS12 acts as a cancer promoter in colorectal cancer via activating the Wnt/β-catenin signaling pathway. Ann Transl Med.

[102] Lu T, Dang S, Zhu R (2017). Adamts18 deficiency promotes colon carcinogenesis by enhancing β-catenin and p38MAPK/ERK1/2 signaling in the mouse model of AOM/DSS-induced colitis-associated colorectal cancer. Oncotarget.

[103] Berendsen AD, Fisher LW, Kilts TM (2011). Modulation of canonical Wnt signaling by the extracellular matrix component biglycan. Proc Natl Acad Sci U S A.

[104] Uribe ML, Marrocco I, Yarden Y (2021). EGFR in Cancer: Signaling Mechanisms, Drugs, and Acquired Resistance. Cancers (Basel).

[105] Maik-Rachline G, Hacohen-Lev-Ran A, Seger R (2019). Nuclear ERK: Mechanism of Translocation, Substrates, and Role in Cancer. Int J Mol Sci.

[106] Guo YJ, Pan WW, Liu SB (2020). ERK/MAPK signalling pathway and tumorigenesis. Exp Ther Med.

[107] Liu YJ, Xu Y, Yu Q (2006). Full-length ADAMTS-1 and the ADAMTS-1 fragments display pro- and antimetastatic activity, respectively. Oncogene.

[108] Cain SA, Mularczyk EJ, Singh M (2016). ADAMTS-10 and -6 differentially regulate cell-cell junctions and focal adhesions. Sci Rep.

[109] Santamaria S, Martin DR, Dong X (2021). Post-translational regulation and proteolytic activity of the metalloproteinase ADAMTS8. J Biol Chem.

[110] Dancevic CM, Fraser FW, Smith AD (2013). Biosynthesis and expression of a disintegrin-like and metalloproteinase domain with thrombospondin-1 repeats-15: a novel versican-cleaving proteoglycanase. J Biol Chem.

[111] Nie J, Dang S, Zhu R (2024). ADAMTS18 deficiency associates extracellular matrix dysfunction with a higher risk of HER2-positive mammary tumorigenesis and metastasis. Breast Cancer Res.

[112] Jiang Y, Yu X, Zhao Y (2021). ADAMTS19 Suppresses Cell Migration and Invasion by Targeting S100A16 via the NF-κB Pathway in Human Gastric Cancer. Biomolecules.

[113] Li T, Zhou J, Jiang Y (2022). The Novel Protein ADAMTS16 Promotes Gastric Carcinogenesis by Targeting IFI27 through the NF-κb Signaling Pathway. Int J Mol Sci.

[114] Ren P, Hughes M, Krishnamoorthy S (2017). Critical Role of ADAMTS-4 in the Development of Sporadic Aortic Aneurysm and Dissection in Mice. Sci Rep.

[115] Kirman DC, Renganathan B, Chui WK (2022). Cell surface nucleolin is a novel ADAMTS5 receptor mediating endothelial cell apoptosis. Cell Death Dis.

[116] Silva SV, Lima MA, Cella N (2016). ADAMTS-1 Is Found in the Nuclei of Normal and Tumoral Breast Cells. PLoS One.

[117] Nidai Ozes O, Mayo LD, Gustin JA (1999). NF-kappaB activation by tumour necrosis factor requires the Akt serine-threonine kinase. Nature.

[118] Xu B, Yuan CW, Zhang JE (2023). Curcumin Inhibits Proliferation of Renal Cell Carcinoma in vitro and in vivo by Regulating miR-148/ADAMTS18 through Suppressing Autophagy. Chin J Integr Med.

[119] Cuffaro D, Ciccone L, Rossello A (2022). Targeting Aggrecanases for Osteoarthritis Therapy: From Zinc Chelation to Exosite Inhibition. J Med Chem.

